# Residents’ Experiences with Personalized Learning in Postgraduate Training; Going Beyond Competency Based Medical Education

**DOI:** 10.5334/pme.2125

**Published:** 2026-06-19

**Authors:** Merel H. de Heer, Efraïm J. Hart, Fedde Scheele, Pim W. Teunissen, Erik W. Driessen

**Affiliations:** 1 Department of education of the faculty of medicine, Vrije Universiteit, Amsterdam, the Netherlands; 2 Department of education of OLVG hospital, Amsterdam, the Netherlands; 3 Faculty of sciences, Athena Institute, Amsterdam the Netherlands; 4 Faculty of sciences, Athena Institute, Vrije Universiteit, the Netherlands; 5 Vrije Universiteit and Universiteit van Amsterdam, in Amsterdam, the Netherlands; 6 Maastricht University, Maastricht, the Netherlands

## Abstract

**Introduction:**

Personalization is increasingly emphasized in postgraduate medical education. While competency-based medical education (CBME) is theoretically positioned to support personalization, practical challenges such as fixed learning outcomes, workplace constraints, time-fixed rotations, and assessment requirements, often limit its realization. This case study aimed to explore how residents experience and make use of individualized development trajectories within a program that combined fixed outcomes with flexible program components.

**Method:**

We conducted a case study using semi-structured interviews and template analysis. The study was situated in the Dutch national Obstetrics and Gynecology residency program, which includes formally embedded individualized development trajectories with open-ended learning goals and no pre-determined assessment. We interviewed 12 residents and discussed their portfolios.

**Results:**

All participants engaged in individualized development trajectories. The level of engagement varied and motivation sprang from: personal interest, moral values and dilemma’s, clinical exposure, role models, and workplace possibilities. Three interacting factors enabled this: 1) formal program structure legitimized and fostered engagement; 2) time and workplace exposure allowed participants to recognize personally meaningful goals; 3) program directors’ support facilitated engagement. Program directors varied in their guidance: some coached, endorsed, or role-modeled; others merely monitored activity; and a few did neither. Participants engaged with the individualized development trajectories, despite the absence of assessment.

**Conclusion:**

Structured autonomy, workplace exposure and personal motivation can support personalized learning in CBME. Personalization requires intentional design, supportive structures, trust towards residents, and coaching attuned to the individual learner.

## 1. Introduction

There is a trend towards more personalized postgraduate medical education (PGME) [1,2]. Personalized learning refers to adapting training to residents’ individual learning needs, interests, and professional development goals [3,4]. Residency programs aim to support personalized learning by focusing on residents’ development as medical experts as well as their personal and professional development [4]. Personal and professional development allows for competence development in a changing healthcare system, where physicians are expected to fulfill diverse roles and adapt to evolving systems [5,6]. Most residency programs use competency based medical education (CBME) with predefined and fixed learning outcomes [7]. However, the use of predefined outcomes may limit opportunities for residents to formulate and pursue personal learning goals [8,9]. To better understand how personalization is addressed within PGME, it is important to consider the role of CBME as the dominant educational approach in PGME.

CBME curricula are typically structured around competency frameworks, such as the CanMEDS competency framework or ACGME Milestones, which define physicians’ competencies beyond medical expertise, including competencies in communication, leadership, health advocacy, scholarship, system-based practice, and collaboration [10,11]. CBME provides an educational framework to support personalization since it focusses on competency-based progression and the tailoring of learning trajectories to individual needs [7]. Individual needs can be met by, for instance, allowing residents to progress at different paces [12,13]. On the other hand, use of fixed learning outcomes in CBME, combined with assessment requirements, and workplace constraints such as time-fixed rotations, may limit opportunities for personalization [14,15]. This creates a contradiction: while personalization is promoted in theory, its practical realization is often limited [16,17]. In response, some programs have introduced more flexible or elective components within training structures, yet there is limited empirical understanding of how such approaches are enacted and experienced by residents [18].

The limited empirical understanding of personalization experience in practice is reflected in literature, where personalization opportunities are primarily described at a conceptual level, while their concrete enactment in PGME remains insufficiently understood [19,20]. It is, therefore, unclear how training programs can balance the use of intended learning outcomes and assessment requirements with opportunities for personalization [21]. To examine this, we conducted a case study within a postgraduate CBME program in obstetrics and gynecology in the Netherlands that includes individualized development trajectories: a part of the training plan that requires residents to focus on broadly defined topics relevant to their future role as gynecologists without fixed learning outcomes or pre-determined assessment, embedded within an otherwise EPA-driven curriculum [22]. This study addressed the following research question: How do residents experience and make use of personalization opportunities within their specialty training program?

## 2. Methods

We conducted a case study with semi-structured interviews to explore residents’ experiences with individualized development trajectories within an existing Dutch obstetrics and gynecology CBME training plan [23,24]. Data were analyzed using template analysis [25], an approach that allows for iterative development of a coding template.

### Setting

The study took place within the Dutch national six-year postgraduate training program for obstetrics and gynecology [22]. The current training plan (National Curriculum for Obstetrics and Gynecology, NCOG [22]) was implemented in 2021. The training plan is competency-based and combines structured EPA-driven training with individualized development trajectories. Residents are required to engage with the individualized development trajectories, but have flexibility regarding the content, timing, and extent of their engagement, in consultation with their program director. The individualized development trajectories are not part of the assessment program and there are no pre-defined learning outcomes. Residents are expected to develop personal learning goals for their individualized development trajectories and to document this development in their digital portfolio. For instance, residents can ask for feedback on activities in daily work, or on dedicated projects related to the individualized development trajectory. Follow-up on their individualized development trajectories is discussed in progress meetings with their program directors. The individualized development trajectories comprise topics relevant to the profession of obstetrics and gynecology. The program provides outlines and examples of how residents, together with their program director, can organize their individualized development trajectories [22]. Table 1 provides an overview of the four topics in individualized development trajectories. Supplement 1 provides more detail on the definition and guidance of individualized development trajectories.

**Table 1:** Individualized Development Trajectory Topics. National Curriculum for Obstetrics and Gynecology (NCOG)*.


**Topic 1**	**Being Engaged, Staying Engaged** Maintaining balance and motivation Self-directed learning Dealing with setbacks

**Topic 2**	**Network Medicine for Specific Target Groups** Women’s Health Care for patients in vulnerable situations

**Topic 3**	**Organization-based Care** Quality, management, safety and sustainability Efficiency and value-driven care Clinical leadership

**Topic 3**	**Knowledge and Innovation** Contributing to and managing change Innovative techniques Education and training Science


*These topics were selected by the National Board of Obstetrics and Gynecology and the curriculum developers, based on their relevance to the Dutch context and the current and future practice of obstetrics and gynecology.

### Data Collection

Email invitations to participate in one-to-one interviews were distributed by local program directors and the national O&G residency association to all residents with at least 3 years of experience with the training program in Dutch training regions. We aimed to include a minimum of 12 participants [26] until no new themes would result from the data analysis. We aimed for a sample with variation in training region and phase of training. All participants provided written informed consent.

All interviews were conducted by a single researcher (MH), who was not involved in residency supervision. The interviews included discussion of participants’ portfolio materials relevant to the individualized development trajectories. The participants were asked to open their digital portfolio during the interview to discuss their entries for development within the individualized development trajectories. Points of discussion were, for example, their logged activities in individualized development trajectories, their personal learning goals, and feedback on those activities.

All sessions (≈45 min) were audio-recorded and transcribed verbatim. Data collection and preliminary analysis proceeded iteratively, allowing early insights and unexpected findings to inform subsequent interviews.

### Data Analysis

Interview data were analyzed using template analysis, following the 7 steps described by King [27]. In step 1 we defined *a priori* themes informed by sensitizing concepts. Transformative learning and self-directed learning (SDL) were used as sensitizing concepts since these are relevant topics in research on personal and professional development in PGME [3,28]. For step 2 we transcribed the interviews and familiarized ourselves with the data by reading each transcript. In step 3 we applied initial coding. The first author (MH) coded the first three transcripts using the coding template. A first analysis of themes followed and was discussed in the full research team. To stimulate discussion, the second author (EH) coded a sample of three interviews separately. The resulting insights from both the first and second author were discussed in the full research team. Coding outcomes and links between codes were compared continuously and discussed until consensus was achieved. Per step 4 and 5, we further developed the coding template, which was discussed in the full research team again. The template guided MH’s coding of the next transcripts. Each interim analysis was presented to the full research team. Team discussions focused on: (i) refining code definitions, (ii) clarifying relationships between codes and themes, and (iii) deciding whether interview prompts needed adjustment to explore emerging ideas. For step 6 we produced the final template that helped interpret and write our results. Data collection and analysis proceeded until theoretical sufficiency was reached, defined as the stage at which: (i) new data fitted within the existing template; (ii) no new insights, perspectives, issues or counter-examples arose in the interviews; and (iii) the team agreed on the final template [29]. Finally, in step 7 all transcripts were reread by MH and EH reviewed the final analysis to ensure that no relevant information had been overlooked. MH kept reflective memo’s, notes and a logbook throughout the complete data collection and analysis process to stimulate researcher reflexivity.

### Team positionality

The research team brought complementary perspectives to the study. MH is a physician and researcher focusing on future-oriented postgraduate medical education within CBME; MH’s experience in CBME curriculum research and prior clinical experience enabled sensitivity to the structural design of training programs and how these are encountered in practice. EH is a physician working clinically in obstetrics and gynecology, with research expertise in transformative learning; EH’s combined expertise in transformative learning research and current clinical practice brought attention to processes of personal and professional development. FS is a scholar of health system innovation and education and dean of a dental faculty. He contributed to a broader system and societal perspective. ED, an educationalist with extensive experience in medical education research but not involved in obstetrics and gynecology training, provided methodological and theoretical distance. PT is a consultant gynecologist and as a curriculum developer involved in the design of the individualized development trajectories of our study setting, contributed insider knowledge of the program’s intentions and structure. These varied positions enabled reflexive discussion throughout template development and interpretation.

### Ethics statement

Ethical approval was granted by the Netherlands Association for Medical Education Ethical Review Board (NVMO-ERB 2024.2.13). The members of the research team worked only with anonymized transcripts. During the interviews with the first author (MH), participating residents logged into their portfolios themselves; access was limited to joint on-screen viewing, and the researcher was not granted independent login credentials.

## 3. Results

12 O&G residents, year 3-6 of residency, from across the Netherlands participated in the study. Interviews were conducted between July 2024 and May 2025. The analysis resulted in three main themes. Each theme is described and illustrated with representative quotes.

### 1. Residents valued development tracks as part of their training

Nearly all participants valued working within the individualized development trajectories for aspects of their professional development not directly related to medical expertise:

‘As part of my professional development, I became increasingly drawn to non-clinical roles, because I enjoy them, and because I realized early on that these tasks are an essential part of our profession, and that we should train in them accordingly.’ – Participant 1

Most residents described that they were not involved in individualized development trajectories out of an obligation to program requirements, but through personally motivated initiatives that the training plan allowed and encouraged:

‘It wasn’t something I did with the idea that it would count towards my individualized development trajectories, I just really wanted to do it.” – Participant 11

Besides these overall experiences, the participants described facilitators and barriers in their personalized learning in two main categories: structured autonomy and motivation through situated engagement.

### 2. Structured autonomy

Structured autonomy was a central theme, referring to the balance between curricular structure that legitimized residents’ engagement with these topics and sufficient openness to allow participants autonomy within the individualized development trajectories. Participants perceived autonomy in their engagement with individualized development trajectories since they could deepen and expand topics on an individual basis. Autonomy was especially evident when structure of the training plan, timing and program director support aligned. This alignment gave participants both permission and direction in their individualized development trajectories, as it legitimized the time spent on these activities, created opportunities for learning and recognition of relevant issues, and allowed program directors to guide them through personal coaching, facilitation, and joint reflection.

#### Structure in the curriculum provides learning support

Participants experienced the program structure and digital portfolio as supportive for working with individualized development trajectories. Participants highlighted that connecting individualized development trajectory work to their digital portfolio was a benefit as some of their work now felt to be a more explicit part of their program. This was despite not being part of the assessment program.

‘Using the portfolio to link my activities to individualized development trajectories, legitimizes the hours I spend’. – Participant 8

‘I was happy that the individualized development trajectories became part of the training plan, and that you could document your activities and development in this area in the portfolio. That gave it value.’ – Participant 2

However, participants did not always recognize how their day-to-day practice included individualized development trajectories. Some explained that they felt it needed to be a big project, especially to be worthy of portfolio documentation. The broadness of the training plan also had the downside of sometimes resulting in abstractness, lacking clarity for participants on how to engage with individualized development trajectories.

‘Hm.. The open nature of this part of the training… I think people need more inspiration about what could be included. I wouldn’t necessarily say you should concretize it in the sense of spoon-feeding it, but I do think you can suggest what some examples might be.’ – Participant 3

#### Timing and exposure shape engagement opportunities

Timing and exposure were critical in shaping engagement. Participants reported focusing primarily on their medical expertise during the early years. As participants became more confident in their clinical skills, they gained space to reflect on broader issues they encountered in practice. The topics chosen in individualized development trajectories helped them to gain new perspectives on what they were experiencing in practice. As residency progressed, the increased exposure to diverse settings and patient populations helped them recognize problems or topics they felt personally compelled to explore.

‘In the first two years, I was focused on clinical content and EPAs; later, I began to recognize the individualized development trajectories.’ – Participant 5

Participants needed sufficient exposure to recognize topics in individualized development trajectories and identify personal learning opportunities, but they could only act on these when circumstances aligned. This alignment depended on the possibilities their rotation site could offer at that moment, as well as whether the timing allowed them to seize opportunities beyond the rotation.

‘Educational culture and improving education are somewhat my mission. The hospital has just merged, so there is a lot to be done and reorganized… You also connect the things you are working on now, where your interests lie, with what you envision for yourself in the future. At the same time, you keep a realistic perspective on what is feasible and appropriate at a given moment.’ – Participant 8

Personal life circumstances also played a role, for instance, becoming a new parent or providing informal care to relatives could temporarily limit the space and energy available for such engagement.

‘I had just had a child, and then it can sometimes be a lot to have to do everything and to engage in an individualized development trajectory’ – Participant 5

#### Program directors’ role in empowerment of autonomy

Program directors had a crucial role in supporting residents’ personalized learning. Participants had different experiences with the way individualized development trajectories were addressed in progress meetings with their program directors. Some facilitated substantive engagement, while others reduced it to formal compliance. Some program directors coached participants on an individual basis, discussing their personal values and interests, alongside their professional development and institutional opportunities. Descriptions and examples of individualized development trajectories by program directors helped clarify what individualized development trajectories meant in practice.

‘My program director has been really important for me… She gave me, in my experience, all the space to reflect on these topics. She very quickly saw and heard from me how important it is to me to be involved in individualized development trajectories. How important that is, in turn, for my sense of engagement… So she really has been a very important factor in that. Yes.’ – Participant 1

Other program directors neither coached nor intervened, leaving individualized development trajectories entirely up to the resident or merely checking for activity without discussing content. This situation created a sense of ticking off a checklist rather than engaging in meaningful dialogue. Participants have an engagement obligation regarding their individualized development trajectories, which should be discussed in the progress reviews with program directors. This could legitimize their time spent on individualized development trajectories according to the participants. However, participants noted that their time was only truly legitimized if program directors actively acknowledged their relevance in meaningful dialogue. If program directors did not discuss development, participants stated that individualized development trajectories could feel like yet another check box, instead of facilitating further personal learning goals and engagement.

‘If your program directors don’t recognize it as part of your training, how are you supposed to develop it (individualized development trajectories) properly?’ – Participant 9

Aligned structure of the training plan, timing and exposure, and program director support enabled autonomy in learning, which facilitated engagement with individualized development trajectories. Participants’ engagement was also dependent on personal motivation and their evolving professional concerns, shaped by what they encountered in clinical practice.

### 3. Motivation through situated engagement

Participants described that their motivation to engage with individualized development trajectories mostly derived from personal interest, moral issues or disorienting dilemmas they experienced during their clinical work. Participants were exposed to different settings, situations and patients. Participants expressed that these different settings and their growing experience in the work field helped them to recognize topics, situations or problems that they found profoundly interesting or troublesome. They often expressed that they came across a situation that (morally) conflicted them, and that their engagement could take the form of small, everyday tasks or more dedicated, larger projects.

This participant described a problem in language barriers that they came across in clinical practice:

‘If we don’t understand a patient’s language, that person is simply ignored. I found that deeply troubling; it just felt wrong…. Part of my drive comes from things I witnessed in my personal life. So first I decided: ‘I’ll request an interpreter for every case. A diversity-lunch talk followed; I dived into culturally sensitive care, gave a presentation, and from there things snowballed…. I have such strong feelings about this, the rest just started rolling.’ – Participant 7

Another participant reflected on prior experience in rural Africa and how that shaped her motivation and feelings today:

‘What motivates me is that I want to keep good healthcare available for every Dutch citizen, and there is a bit of background to that of course, because I have worked in rural Africa where there was a huge difference between wealthy people and those who were less educated and less privileged in their access to care. So I also feel the need to make more of a medium-term impact and to be able to care for patients better next week than this week’ – Participant 2

Some participants described changes in how they viewed their profession, describing perspective changes based on their learning experiences in the individualized development trajectories:

‘Actually, in all the answers, I think that it is very clearly a different perspective. Yes, again, I think I didn’t even have a perspective on it when I started, and now I do, and that perspective will continue to change.’ – Participant 1

‘I think very differently about the organization of care. I have become much more aware of how you could bring about certain projects or cultural changes. I have become much more aware and have learned a lot about how to, well, think more strategically, and how you could take on an innovation.’ – Participant 6

With almost all participants, motivation sprang from dilemmas that resonated with participants’ values. The first years, interacting with all four topics made them aware of relevant areas for their profession that were not directly related to medical expertise. That primed them to recognize overarching topics, problems and dilemmas resonating with their values in day-to-day practice.

‘Working with individualized development trajectories improves the engagement for your profession, since you can include your own values, interests and contribution to clinical practice. That drives me!’. – Participant 6

Participants often stated that they did not work with the deepening and expanding part of individualized development trajectories simply because it was part of the training plan, rather they worked on it from intrinsic motivation:

‘I would have done this anyway; Individualized development trajectories just give it a name.’ – Participant 4

## 4. Discussion

In this case study we explored obstetrics and gynecology residents’ experiences with personalization opportunities within their specialty training. Residents engaged in individualized development trajectories without predefined outcomes or formal assessment. We observed what we called structured autonomy, which reflected a balance between sufficient curricular structure to legitimize engagement, supported by clinical exposure and program director guidance, and sufficient openness to allow autonomy. Residents stated that individualized development trajectories added value in training for their future profession. The level of engagement differed amongst residents, and motivation to engage was shaped by residents’ personal interests in combination with exposure to clinical practice.

A leading principle in medical education is that assessment drives learning [30]. This principle suggests that learners engage primarily when the content is assessed [31,32]. CBME has also been shaped by this principle and relies on assessment programs that frequently assess, often fixed and universal, outcomes [33,34]. However, our findings show learners’ engagement with learning content despite the absence of summative assessment. All participants were actively engaged with individualized development trajectories and showed ongoing personalized learning. Their engagement seemed to be driven by personal interest, workplace experiences, and longitudinal reflection. Key to this engagement was the ‘structured autonomy’ that the participants experienced in the individualized development trajectories. The individualized development trajectories offered autonomy for choosing topics that matched with personal interests and situations, and autonomy for how to work on these topics. International research on workplace-based learning provides wider lenses, next to assessment drives learning [35]. Authors state that learning happens all the time, implicitly and explicitly, since the workplace context provides learning in the moment, but in time it also provides professional and personal development through exposure to different contexts and cases [36,37,38]. This aligns with our results, where structured autonomy may also drive learning: when learners are offered freedom within a clearly supported framework, it creates both opportunity and context, as well as space for autonomy and personalization.

Transformative learning (TL) offers a useful framework for understanding the motivation that residents experience when engaging with individualized development trajectories. TL is a process of lasting change in a learners’ perspective, beliefs, and values, strengthening their ability to act as agents of change in practice [3,39]. It involves not only gaining knowledge and skills, but also rethinking how they see themselves, their roles, and their place within the healthcare system. Through critical reflection on disorienting dilemmas, residents may improve their clinical practice [9]. From a social change perspective, TL can also enable residents to identify necessary innovations, systemic shortcomings, or injustices in healthcare, and to act accordingly [5,6]. Residents expressed that the freedom afforded in individualized development trajectories allowed them to reflect on their professional practice and personal values, enabling them to adopt new perspectives and initiate actions, consistent with elements of TL. In medical education, implementing transformative learning is challenging, as it often occurs implicitly and goes unrecognized, while programs and hidden curricula prioritize the medical expert role [6,40]. Our findings suggest that a program with partially open and personalized components can foster TL processes for residents and make TL more visible and systematic.

Personalization enables residents to develop competencies in alignment with their individual interests and professional development [41]. While CBME is intended to facilitate such personalized training, in practice it often operates within rigid structures due to fixed learning outcomes and assessment programs, which may inadvertently constrain the flexibility it aims to promote [17,42]. Our findings showed that the participants engaged meaningfully with the personalized components without being part of the assessment program or predefined learning outcomes in the combined approach of our study setting. The context was important in this study since the O&G training program has over 20 years of experience with CBME [2]. Over this period, it has advanced through iterative implementation of three CBME-based training plans, informed by learner and faculty feedback, and supported by a national quality assurance system [2,43,44]. Each plan emphasized faculty development and educational leadership and built on lessons learned whilst aligning with new insights from medical education literature [2,16,45]. To implement similar interventions in other contexts, comparable enabling mechanisms may be required, including sustained investment in faculty development, educational leadership, and a coherent quality assurance system. Practical recommendations derived from our study are summarized in Table 2.

**Table 2:** Practical recommendations to inform program design and workplace practices.


PRACTICE POINT	RECOMMENDATION

**EPAs are essential, but not the full story**	Combine clearly defined EPAs with space for broader, personalized learning. This allows residents to engage with the wider scope of the specialty.

**Allow time for exposure and growth**	Ensure early training focuses on clinical expertise, with time to encounter diverse situations that later prompt deeper, personal engagement with individualized development trajectories.

**Avoid over-structuring with assessments**	Create learning spaces without checklists or summative assessment. Residents described these as enabling autonomy, reflection, and even transformation. Such open spaces enable transformative learning by prompting reflection that reshapes perspectives.

**Open-ended goals can work alongside EPAs**	In a dual approach program, open-ended goals can be effective. Provide examples and role models to guide residents in navigating these more abstract domains.

**Coaching is not one-size-fits-all**	Support program directors in tailoring their coaching. Residents valued when program directors offered personalized guidance/coaching and legitimized individualized development trajectories.


### Limitations and future research

This study examined residents’ experiences with a distinctive component of their specialty training: individualized development trajectories that are not part of the assessment program or predefined learning outcomes. This setting offered a valuable opportunity to explore learning beyond assessment-driven behavior, whilst also potentially limiting transferability of our findings since they are grounded in a single specialty and national context. However, the resident experience and the training plan have potential value for program designers in other medical specialties and in other countries as well. This relevance extends beyond our specific context, as CBME is the dominant approach in PGME worldwide [1] and residency training, being workplace-based, inherently provides opportunities for learning without predefined outcomes, as clinical practice itself continuously presents situations from which residents can form personal learning opportunities. Therefore, we expect our results to be helpful to other contexts, for example in the (re)design of PGME curricula.

In addition, our analysis focused solely on the learner perspective. Residents offered practice-based experience and reflections on their development, which is an essential perspective in understanding if and how learning takes place in this setting. Further insight could be gained by exploring how program directors interpret, support and observe this form of learning.

While our study highlights perceived engagement and learning, we did not evaluate the educational effectiveness of the training program, nor did we quantify engagement. In our sample, we included different Dutch regions to prevent regional bias, and we reached out to all residents of O&G who had at least a few years’ experience with the program. It is possible that residents who were not at all engaged with their development trajectories were reluctant to participate in this study. Finally, we did not examine intersectional factors in this study, although these may influence residents’ personal motivation and values. Future research could reflect factors, such as gender, age, ethnicity, culture, economic backgrounds and health.

## Conclusion

This study indicated that structured autonomy allowed residents to engage actively in personalized learning opportunities, without pre-defined learning goals and without being part of the formal assessment program. Their motivation was based on clinical experience, personal interests and support from their program director. These findings suggest that combining predefined outcomes with individualized learning opportunities can support greater personalization in PGME. Personalization and flexibility in training plans, combined with intrinsic motivation can be more fully leveraged for personal and professional development in PGME.

## Additional Files

The additional files for this article can be found as follows:

10.5334/pme.2125.s1 Supplement 1. Details of individualized development trajectories.

10.5334/pme.2125.s2 Supplement 2. Interview guide.

10.5334/pme.2125.s3 Supplement 3. Templates.

## References

[1] ten Cate O. Competency-Based Postgraduate Medical Education: Past, Present and Future. GMS Journal for Medical Education. 2017;34. 10.3205/zma001146 PMC5704607 29226237

[2] de Heer MH, Driessen EW, Teunissen PW, Scheele F. Lessons learned spanning 17 years of experience with three consecutive nationwide competency based medical education training plans. Frontiers in Medicine. 2024;11. DOI: 10.3389/fmed.2024.1339857 PMC10917951 38455473

[3] Frenk J, Chen L, Bhutta ZA, Cohen J, Crisp N, Evans T, et al. Health professionals for a new century: transforming education to strengthen health systems in an interdependent world. Lancet. 2010;376(9756):1923–1958. DOI: 10.1016/S0140-6736(10)61854-5 21112623

[4] Hodges BD. A tea-steeping or i-Doc model for medical education? Acad Med. 2010;85(9 Suppl):S34–S44. DOI: 10.1097/ACM.0b013e3181f12f32 20736582

[5] Frenk J, Chen L, Michaud C. The future of health professional education. In: Walsh K, editor. Oxford Textbook of Medical Education: Oxford University Press; 2013. DOI: 10.1093/med/9780199652679.003.0059

[6] Hart EJ, de Heer-Koster MH, van der Harst M, Browne JL, Scheele F. Key tips to shift student perspectives through transformative learning in medical education. BMC Med Educ. 2025;25(1):202. DOI: 10.1186/s12909-025-06754-2 39920665 PMC11806662

[7] Van Melle E, Frank JR, Holmboe ES, Dagnone D, Stockley D, Sherbino J, et al. A Core Components Framework for Evaluating Implementation of Competency-Based Medical Education Programs. Acad Med. 2019;94(7):1002–1009. DOI: 10.1097/ACM.0000000000002743 30973365

[8] van Loon K, Teunissen P, Driessen E, Scheele F. The Role of Generic Competencies in the Entrustment of Professional Activities: A Nationwide Competency-Based Curriculum Assessed. Journal of Graduate Medical Education. 2016;8. DOI: 10.4300/JGME-D-15-00321.1 PMC5058587 27777665

[9] Scheele F, Van Luijk S, Mulder H, Baane C, Rooyen CD, De Hoog M, et al. Is the modernisation of postgraduate medical training in the Netherlands successful? Views of the NVMO Special Interest Group on Postgraduate Medical Education. Med Teach. 2014;36(2):116–120. DOI: 10.3109/0142159X.2013.849333 24256088

[10] Frank JR, Snell L, Sherbino J. CanMEDS 2015 Physician Competency Framework. Royal College of Physicians and Surgeons of Canada; 2015.

[11] Celeste Eno P, Ricardo Correa MDE, Nancy H, Stewart D, Jonathan Lim M, Mary Elizabeth Westerman M, Eric S, Holmboe M, et al. Milestones Guidebook for Residents and Fellows. Accreditation Council for Graduate Medical Education (ACGME); 2020.

[12] ten Cate O, Scheele F. Competency-Based Postgraduate Training: Can We Bridge the Gap between Theory and Clinical Practice? Acad Med; 2017. 10.1097/ACM.0b013e31805559c7 17525536

[13] Walsh K. The future of postgraduate training. Pan Afr Med J. 2014;19:333. DOI: 10.11604/pamj.2014.19.333.5555 25918573 PMC4405066

[14] Touchie C, ten Cate O. The promise, perils, problems and progress of competency-based medical education. Med Educ. 2016;50(1):93–100. DOI: 10.1111/medu.12839 26695469

[15] Birman NA, Vashdi DR, Miller-Mor Atias R, Riskin A, Zangen S, Litmanovitz I, et al. Unveiling the paradoxes of implementing post graduate competency based medical education programs. Med Teach. 2025;47(4):622–629. DOI: 10.1080/0142159X.2024.2356826 38803298

[16] van Rossum TR, Scheele F, Sluiter HE, Paternotte E, Heyligers IC. Effects of implementing time-variable postgraduate training programmes on the organization of teaching hospital departments. Med Teach. 2018;40(10):1036–1041. DOI: 10.1080/0142159X.2017.1418850 29385864

[17] Boyd VA, Whitehead CR, Thille P, Ginsburg S, Brydges R, Kuper A. Competency-based medical education: the discourse of infallibility. Med Educ. 2018;52(1):45–57. DOI: 10.1111/medu.13467 29076231

[18] Darbyshire D, Baker P, Agius S, McAleer S. Trainee and supervisor experience of the Academic Foundation Programme. J R Coll Physicians Edinb. 2019;49(1):43–51. DOI: 10.4997/jrcpe.2019.111 30838993

[19] Frank JR, Snell LS, Oswald A, Hauer KE, International CC. Further on the journey in a complex adaptive system: Elaborating CBME. Med Teach. 2021;43(7):734–736. DOI: 10.1080/0142159X.2021.1931083 34097832

[20] Van Schalkwyk SC, Hafler J, Brewer TF, Maley MA, Margolis C, McNamee L, et al. Transformative learning as pedagogy for the health professions: a scoping review. Med Educ. 2019;53(6):547–558. DOI: 10.1111/medu.13804 30761602

[21] Szulewski A, Braund H, Dagnone DJ, McEwen L, Dalgarno N, Schultz KW, et al. The Assessment Burden in Competency-Based Medical Education: How Programs Are Adapting. Acad Med. 2023;98(11):1261–1267. DOI: 10.1097/ACM.0000000000005305 37343164

[22] NVOG. NCOG National Curriculum for Obstetrics and Gynecology 2021 [Available from: https://nvog-logo.nl/wp-content/uploads/2024/01/LOGO_fullEN_version921_1223.pdf.

[23] Yin RK. Case study research and applications : design and methods. 6 ed. Thousand Oaks, California: SAGE; 2018. p. 453.

[24] Schwarz TB, Reiling C. Preparing an Interpretive Research Design – Chapter 9 In: Jennifer Eb, Goodman CaSW, editors. Doing good qualatative research. Oxford University Press; 2024. DOI: 10.1093/oso/9780197633137.003.0009

[25] Brooks J. The Utility of Template Analysis in Qualitative Psychology Research. Qualitative Research in Psychology. 2015. DOI: 10.1080/14780887.2014.955224 PMC4960514 27499705

[26] Wutich A, Beresford M, Bernard HR. Sample Sizes for 10 Types of Qualitative Data Analysis: An Integrative Review, Empirical Guidance, and Next Steps. International Journal of Qualitative Methods. 2024;23. DOI: 10.1177/16094069241296206

[27] King N. Doing Template Analysis. In: Symon G, Cassell C, editors. Qualitative Organizational Research: Core Methods and Current Challenges: Sage Academic Books; 2012. DOI: 10.4135/9781526435620.n24

[28] Wilkes S, Maggio LA, Martin PC, Melton J, Zheng B. Self-Directed Learning in Health Professions Education: A Systematic Review and Meta-Analysis. Perspect Med Educ. 2026;15(1):37–52. DOI: 10.5334/pme.2128 41626409 PMC12857615

[29] Bowen GA. Naturalistic inquiry and the saturation concept: a research note. Qualitative Research. 2008;8(1):137–152. DOI: 10.1177/1468794107085301

[30] Wormald BW, Schoeman S, Somasunderam A, Penn M. Assessment drives learning: an unavoidable truth? Anat Sci Educ. 2009;2(5):199–204. DOI: 10.1002/ase.102 19743508

[31] Newble DI, Jaeger K. The effect of assessments and examinations on the learning of medical students Medical Education. 1983;17:165–171. DOI: 10.1111/j.1365-2923.1983.tb00657.x 6865814

[32] Ganesan I, Cham B, Teunissen PW, Busari JO. Stakes of Assessments in Residency: Influence on Previous and Current Self-Regulated Learning and Co-Regulated Learning in Early Career Specialists. Perspect Med Educ. 2023;12(1):237–246. DOI: 10.5334/pme.860 37334108 PMC10275342

[33] Cilliers FJ, Schuwirth LW, Adendorff HJ, Herman N, van der Vleuten CP. The mechanism of impact of summative assessment on medical students’ learning. Adv Health Sci Educ Theory Pract. 2010;15(5):695–715. DOI: 10.1007/s10459-010-9232-9 20455078 PMC2995206

[34] van der Vleuten CP, Schuwirth LW, Driessen EW, Dijkstra J, Tigelaar D, Baartman LK, et al. A model for programmatic assessment fit for purpose. Med Teach. 2012;34(3):205–214. DOI: 10.3109/0142159X.2012.652239 22364452

[35] Kusurkar RA, Orsini C, Somra S, Artino AR Jr., Daelmans HEM, Schoonmade LJ, et al. The Effect of Assessments on Student Motivation for Learning and Its Outcomes in Health Professions Education: A Review and Realist Synthesis. Acad Med. 2023;98(9):1083–1092. DOI: 10.1097/ACM.0000000000005263 37146237 PMC10453393

[36] Martin L, Blissett S, Johnston B, Tsang M, Gauthier S, Ahmed Z, et al. How workplace-based assessments guide learning in postgraduate education: A scoping review. Med Educ. 2023;57(5):394–405. DOI: 10.1111/medu.14960 36286100

[37] Knowles M p, paperbound. (1977). Self-Directed Learning: A Guide For Learners And Teachers. New York: Association Press; 1975.

[38] Sawatsky AP, Ratelle JT, Bonnes SL, Egginton JS, Beckman TJ. A model of self-directed learning in internal medicine residency: a qualitative study using grounded theory. BMC Med Educ. 2017;17(1):31. DOI: 10.1186/s12909-017-0869-4 28148247 PMC5288975

[39] Mezirow J. Transformative Learning: Theory to Practice. New Directions for Adult and Continuing Education. 2002;1997(74):5–12. DOI: 10.1002/ace.7401

[40] Vipler B, Knehans A, Rausa D, Haidet P, McCall-Hosenfeld J. Transformative Learning in Graduate Medical Education: A Scoping Review. J Grad Med Educ. 2021;13(6):801–814. DOI: 10.4300/JGME-D-21-00065.1 35070093 PMC8672835

[41] Holmboe ES. The Transformational Path Ahead: Competency-Based Medical Education in Family Medicine. Fam Med. 2021;53(7):583–589. DOI: 10.22454/FamMed.2021.296914 34000051

[42] Holmboe ES, Sherbino J, Englander R, Snell L, Frank JR, Collaborators I. A call to action: The controversy of and rationale for competency-based medical education. Med Teach. 2017;39(6):574–581. DOI: 10.1080/0142159X.2017.1315067 28598742

[43] Taber S, Akdemir N, Gorman L, van Zanten M, Frank JR. A “fit for purpose” framework for medical education accreditation system design. BMC Med Educ. 2020;20(Suppl 1):306. DOI: 10.1186/s12909-020-02122-4 32981517 PMC7520946

[44] Nousiainen M, Scheele F, Hamstra SJ, Caverzagie K. What can regulatory bodies do to help implement competency-based medical education? Med Teach. 2020;42(12):1369–1373. DOI: 10.1080/0142159X.2020.1809640 32847447

[45] Van der Lee N, Fokkema J, Westerman M, Driessen E, Van der Vleuten C, Scherpbier A, et al. The CanMEDS framework: Relevant but not quite the whole story. Medical teacher. 2013;35. DOI: 10.3109/0142159X.2013.827329 24003989

