# Supplementary material for: Residents’ Experiences with Personalized Learning in Postgraduate Training; Going Beyond Competency Based Medical Education
Source: Perspect Med Educ. 2026 Jun 19;15(1):523–33. doi: 10.5334/pme.2125 (PMC13281732; doi:10.5334/pme.2125)
Supplement: Supplement 1. — Details of individualized development trajectories. [file pme-15-1-2125-s1.pdf]

Residents’ Experiences with Personalized Learning in Postgraduate Training; Going Beyond Competency Based Medical Education

Supplement 1: Definition and Guidance Individualized Development Trajectories NCOG

Source: nvog-logo.nl/wp-content/uploads/2024/01/LOGO_fullEN_version921_1223.pdf

Definition Individualized Development Trajectories

Individualized development trajectories in NCOG are used to legitimize topics that deserve attention in obstetrics and gynecology education. Individualized development trajectories are descriptions of subjects in which residents can further develop sophisticated aspects of work-related competences and pay attention to personal and professional development. Attention to both EPAs and Development track are necessary to educate gynecologists who can contribute to the best possible care for women in all stages of life now and in the future. Of course, topics within individualized development trajectories often also require specific knowledge and skills, just like EPAs. NCOG provides guidance for education within individualized development trajectories. It also leaves ample room for interpretation in line with the interests of individual residents and the possibilities offered by specific educational institutions. Within each theme there are a number of relevant topics. Residents and program directors are expected to pay attention to all individualized development trajectories and topics at a basic level. In addition, there is room for in-depth study of a topic or for individual topics within these deliberately broadly chosen individualized development trajectories. Residents and program directors have the freedom to determine the education activities they use to flesh out the individualized development trajectories themselves

In terms of content, the topics that require attention can be divided into four categories, which are:

- Being Engaged, Staying Engaged Topics: Maintaining balance and motivation Self-directed learning Dealing with setbacks
- Network medicine for specific target groups Topics: Women's Health Care for patients in vulnerable situations
- Organization-based care Topics: Quality, management, safety and sustainability Efficiency and value-driven care Clinical leadership

- Knowledge and innovation Topics: Contributing to and managing change Innovative techniques. Education and training, Science

The individualized development trajectories are described in more detail in publicly available training plan. They are based on the past training plans, vision documents on medical (specialist) care in the future and scientific literature on the content and form of health profession education. Individualized development trajectories appear throughout education. They can be recognized in the vast majority of the work of residents and gynecologists. It is the explicit intention to pay attention to both EPAs and individualized development trajectories in daily work.

Often, during feedback moments, aspects of one or more EPAs and individualized development trajectories can be chosen as learning objectives and be the subject of interaction between resident and supervisor. Individualized development trajectories can be supported by various forms of education. These should be targeted and deliberately used to make learning in practice more efficient or to deepen it. Each topic of the four individualized development trajectories should receive attention at a basic level. Later in the education there will be room for more in-depth coverage of selected topics or for the addition of topics. This provides opportunities for individual interpretation by residents or within local or regional education programs. Regional and local programs should work out how the deliberately broadly chosen topics are fleshed out and how space is provided for individually chosen topics. This can be done with educational activities that have their origin in the workplace and/or with education activities such as a course, training or targeted project. In addition, other forms of guidance can be considered, such as coaching or InterVision. For example, residents can also contribute to development and improvement projects in a healthcare institution or region on topics that they are required to pay attention to or that they select together with the program director. NCOG recommends some working forms and suggests many possible working forms.

Guidance within Individualized Development Trajectories

Individualized development trajectories are mainly (but not only) about supervision of development in topics for which no universal learning outcomes can be agreed upon. Nevertheless, an individual resident can discuss with the program director what developmental goals they have for specific topics and individualized development trajectories, and how development towards these goals can be supported and monitored. Feedback on development within individualized development trajectories will then often involve openness and self-reflection on the part of the resident and the supervising program director. To facilitate this conversation and create some form of transparency around development within individualized development trajectories, a resident is asked to

occasionally reflect on the following questions (and to record this in the portfolio) for each topic:

- inspiration: what was the reason to pay attention to this topic and what development was sought?
- effort: what is/has been done in terms of development on this topic?
- impact: what impact do the efforts have on the development of the residents and/or on the working environment?

These aspects can be illustrated in the portfolio in a variety of ways. Residents are given freedom in this regard. Of course, it is also possible to use feedback forms that are incorporated in the portfolio and link these to a topic within a theme.

Examples of ways to record effort and impact are:

- Reflection report
- 360-degree feedback
- Published paper
- Project description or report
- Letter from a patient about her experience of care receive
