# Supplementary material for: Residents’ Experiences with Personalized Learning in Postgraduate Training; Going Beyond Competency Based Medical Education
Source: Perspect Med Educ. 2026 Jun 19;15(1):523–33. doi: 10.5334/pme.2125 (PMC13281732; doi:10.5334/pme.2125)
Supplement: Supplement 2. — Interview guide. [file pme-15-1-2125-s2.pdf]

SEMI-STRUCTURED INTERVIEW GUIDE

Residents experience with individualized development trajectories

Introduction

Thank you for participating in this study. The aim of this interview is to gain a better understanding of how residents experience the individualized development trajectories in their training. There are no right or wrong answers to our questions; we are interested in your own experiences. Participation in this study is voluntary, and your decision to participate or not will not affect your training progress. The interview will take approximately 45 minutes. With your permission, I would like to record the interview because I do not want to miss any of your comments. All responses will be treated confidentially. You may always refuse to answer a question or stop the interview at any time and for any reason. Do you have any questions about what I have just explained or before we begin the interview?

May I start the recording?

Topic List

1. Exploration
2. Experience with individualized development trajectories
3. Effort invested in individualized development trajectories
4. Follow-up of individualized development trajectories: discussing the portfolio
5. Context
6. Closing

Model

The following theoretical frameworks informed the interview guide:

- Transformative learning
- Self-directed learning

Interview 1. Exploration

At what stage of the training are you currently, and where are you currently completing your training?

Before we start the substantive questions, I would like to briefly explain the definition of the individualized development trajectories in the national training plan:

“Alongside EPAs, explicit attention is given to the personal and professional development of residents. Individualized development trajectories describe topics that are important for gynecologists to contribute—now and in the future—to the best possible care for women across all life stages. These include topics such as work engagement, dealing with errors, learning (self-directed learning), contributing to innovation in care, and scientific activities. LOGO describes four individualized development trajectories: being engaged and sustaining engagement; network medicine (women’s health, the vulnerable patient); organization-related care; and knowledge and innovation.”

2. Experience with individualized development trajectories

I would like to start the interview section about individualized development trajectories with an open question:
How have you experienced the individualized development trajectories in your training?

Possible follow-up questions:

- Could you explain that a bit more?
- What do you mean by that?
- Why do you think that is?

What is the purpose of the individualized development trajectories in the training program? And how does this relate to your own training?

Possible follow-up questions:

- How do the individualized development trajectories contribute to your training?
- What do you think you gain from them during your training?
- What motivates you within the individualized development trajectories?
- How do the individualized development trajectories contribute to a sense of meaning in your training?

If the individualized development trajectories did not exist, what would you miss? Has this changed during your training?

---

3. Specific individualized development trajectories

1. Have you already worked on a specific individualized development trajectory?
   What do you associate with this individualized development trajectory?
   How does it appear in the training?

If not, why not?
Follow-up questions:

- Have you perhaps worked on topics related to the individualized development trajectories in another way?
- Is it possible that you have engaged with the topic but not explicitly within the framework of the individualized development trajectories in LOGO?

If not, are there perhaps indirect topics you are working on that relate to this individualized development trajectory?

2. Have you identified a dilemma or problem that you wanted to address?
   If yes:

- How did you feel about that?

- What plans do you have for the (further) development of this individualized development trajectory?
- How do you weigh your choices?
- With whom have you discussed this?
- What role does your supervisor play in this?

---

4. Effort invested in individualized development trajectories

How do you experience the effort you invest in the individualized development trajectories?

- How much time do you spend on them?
- What does this effort mean to you?
- What motivates you?
- What hinders you?

How do you monitor your development within the individualized development trajectories?

Possible follow-up questions:

- Could you explain that further?
- What do you mean by that?

Are there aspects you would like to change within your individualized development trajectory or dilemma? What prevents you from doing so?

How has your effort in the individualized development trajectories evolved during your training? How have you developed within the individualized development trajectory of network medicine and women's health?

Discussion of the national and local training plan:
How are the individualized development trajectories implemented within your training cluster?
Do you receive time within your training to work on them?

---

5. Follow-up of individualized development trajectories: discussing the portfolio

- Are individualized development trajectories discussed during your progress meetings with your supervisor?
- What kinds of things do you document in your portfolio?
- I would like to review your portfolio together to see how the individualized development trajectories appear in it. Based on your portfolio, we will discuss this further.

Ask the resident to log in.

Based on the content of the portfolio and the presence or absence of individualized development trajectories, discuss this further.

Discuss the learning history based on the portfolio:

- What have you personally learned?
- What have you learned in the context of your training?
- What have you learned in relation to societal issues?

Have there been moments of new insights or perspective changes? Has your perspective on the individualized development trajectories changed?

How could change within your individualized development trajectory or dilemma be achieved during your training?
Have you already made attempts to do so?

---

6. Context

Depending on the stage of training:

General hospital context:

- How are the individualized development trajectories addressed in practice?
- Do your supervisors refer back to them?
- Are there role models within the department regarding the topics of the individualized development trajectories?
- Are they discussed with peers, nurses, midwives, or others?

Academic context:

- How are the individualized development trajectories addressed in practice?
- Do your supervisors refer back to them?
- Are there role models within the department regarding the topics of the individualized development trajectories?
- Are they discussed with peers, nurses, midwives, or others?

Do you perceive differences in how individualized development trajectories are addressed in academic versus general hospitals?

---

7. Closing

Is there anything else you would like to discuss that has not yet been addressed during this interview?

Thank you for your time and participation.
