# Supplementary material for: Residents’ Experiences with Personalized Learning in Postgraduate Training; Going Beyond Competency Based Medical Education
Source: Perspect Med Educ. 2026 Jun 19;15(1):523–33. doi: 10.5334/pme.2125 (PMC13281732; doi:10.5334/pme.2125)
Supplement: Supplement 3. — Templates. [file pme-15-1-2125-s3.pdf]

Final template: these themes are broadened with code groups below

Themes	What?	Description

Initial template

Themes	What?	Where?	Code groups	Codes	Memo's
Resident experience	RQ	RQ & interview guide			
		General experience			
		Motivation			
		Facilitators and barriers			
		Portfolio			
		Support and follow-up by program directors			
		Context			
Self-directed learning	Theoretical background	Value personal competence			
		Formulating Personal learning goals			
		Resources and personal learning activities			
		Obtain feedback on activities			
		Self-reflection --> Value personal competence			
Transformative learning	Theoretical background	Informative, formative and transformative learning			
		Disorienting/moral dilemma and critical reflection			
		Perspective transformation			
		Critical reasoning and system thinking			
		Medical expert --> Medical professional --> Change agent			

Coaching & Structure	Work-culture and context	Influences resident experience	The workculture and context influence how residents can work with themes
		Trainer	Can facilitate, coach, be strict or not ask about it at all
		work culture	Rolmodels in themes, facilitation on workfloor in time & opportunity
		opportunities in the hospital	Depends what projects and vision the hospital has and what opportunities can be there
			Also if a hospital will allow your own projects
		Portfolio	Portfolio helped in this curriculum element according to residents
Motivation	Assessment		
		Instrinsic motivation	Elements of SDL are there, personal learning goals, intrinsic motivation and reflection
			Explicit formulation of learning goals is not something that came along often
		No assessment on themes	For most it would hinder their process if there was assessment
			For some it may have pushed a bit more for some collegues
			Most would say you can't even assess these themes in a constructive way
Timing & exposure	Time-line		
		Theoretical background	10 steps of Mezirow, some showed all, some showed elements of TL
		1-2 year	Focus on medical content, learning about themes --> Mostly medical expert
		3-4 year	Focus shifting, more room for personal interests --> Getting to know their role as gyn, professional
		5-6 year	Personalized plan in differentiation --> Going a step further: what kind of gyn will I be: change agent
Legitimatio n	Individualized development trajectories		
		Legitimation of work	Residents don't work from themes, but from personal interest. Curriculum legitimates this work
		Understanding of curriculum	Interpretation of and work in this curriculum element differs
Autonomy and medical safety	EPA's and individualized development trajectories		
		EPA's are medical precondition	EPA's are often called "trics" you have to learn, they safeguard patient safety
			As soon as medical content is "in control", there is room for personal interest and themes
		Professional development	Residents viewed their individualized development trajectories as important preparation for their actual profession
		Individualized development trajectories are personal	Residents expressed gratitude for the autonomy, they expressed autonomy as motivation

Final themes for formation of results section

1. Residents value individualized development trajectories in residency training

Individualized development trajectories within the curriculum

- 2.1 Residents rarely frame activities explicitly as “working on individualized development trajectories”
  Residents often engage in activities related to themes without explicitly conceptualizing these as individualized development trajectories.
- 2.2 Engagement is largely driven by intrinsic motivation
  Residents describe pursuing activities related to themes primarily based on personal interests and motivations.
- 2.3 The curriculum structure legitimises engagement with themes
  The presence of individualized development trajectories in the curriculum provides legitimacy and space for residents to pursue these interests.
- 2.4 Understanding of individualized development trajectories varies among residents
  - 2.4.1 Some residents were uncertain what individualized development trajectories entailed
  - 2.4.2 Some initially perceived individualized development trajectories as a checklist or obligation
- 2.5 Diversity in interests and backgrounds shapes engagement
  Residents describe a wide range of individual interests and activities related to themes.

---

2. Structured autonomy

Structured autonomy refers to the balance between curricular structure that legitimizes engagement with individualized development trajectories and sufficient openness to allow individual direction within the elective space.

I. Structure: legitimizing yet potentially abstract

- 2.3 The curriculum structure legitimises engagement with themes
- 5.1 Residents express ambivalence toward stricter assessment of individualized development trajectories
  - 5.1.1 Stricter assessment could undermine intrinsic motivation
  - 5.1.2 Some residents note that minimal expectations can function as a nudge
- 5.2 Individualized development trajectories address areas not easily captured in EPAs
- 5.3 Residents nevertheless engage with themes despite limited formal assessment

    - 5.3.1 This aligns with the principle that assessment influences learning

---

II. Timing and exposure shape engagement

Development over time during residency

- 1.1 Early years: focus on medical competence
  Residents describe the first years of training as primarily focused on acquiring medical knowledge and procedural skills.
- 1.2 Middle years: exploring personal interests
  As residents gain confidence in clinical work, they begin to explore personal interests within individualized development trajectories.
- 1.3 Final years: deepening and focus
  In later years, residents describe a clearer focus and deeper engagement with specific themes.
- 1.4 Development over time creates space for broader learning
  The progression of clinical competence over time creates space for engagement with themes and for broader professional development.

Relationship between EPAs and individualized development trajectories

- 3.1 EPAs and individualized development trajectories function as parallel curriculum structures
- 3.2 EPAs form the medical foundation of training
    - 3.2.1 Residents often describe EPAs as technical or procedural tasks
    - 3.2.2 Early training is strongly oriented toward achieving EPA competence
- 3.3 Individualized development trajectories enable deeper professional development
  Individualized development trajectories allow residents to engage with broader professional roles beyond technical competence.
- 3.4 Space for individualized development trajectories emerges after medical competence increases
    - 3.4.1 Residents report limited space for themes in early years
    - 3.4.2 As clinical competence grows, space emerges for engagement with themes

---

III. Program director support

Workplace culture and context

- 4.1 Workplace culture influences opportunities to engage with themes
- 4.2 Culture shapes both opportunities and role modelling

    - 4.2.1 Opportunities and encouragement to pursue interests
    - 4.2.2 Role models demonstrating engagement with themes
- 4.3 Local working culture matters more than hospital type

---

3. Motivation through situated engagement

- 2.2 Engagement is largely driven by intrinsic motivation
- 2.5 Diversity in interests and backgrounds shapes engagement
- 5.4 Elements of self-directed learning are present
    - 5.4.1 Intrinsic motivation and reflection are visible
    - 5.4.2 Explicit goal setting and external guidance are less prominent
